# Infiltration of Immunoinflammatory Cells and Related Chemokine/Interleukin Expression in Different Gastric Immune Microenvironments

**DOI:** 10.1155/2020/2450569

**Published:** 2020-12-23

**Authors:** Ang Wang, Siru Nie, Zhi Lv, Jing Wen, Yuan Yuan

**Affiliations:** ^1^Tumor Etiology and Screening Department of Cancer Institute and General Surgery, The First Hospital of China Medical University, Shenyang 110001, China; ^2^Key Laboratory of Cancer Etiology and Prevention in Liaoning Education Department, The First Hospital of China Medical University, Shenyang 110001, China; ^3^Key Laboratory of GI Cancer Etiology and Prevention in Liaoning Province, The First Hospital of China Medical University, Shenyang 110001, China

## Abstract

Gastric mucosal immune microenvironment plays an important role in the occurrence and development of diseases such as inflammation and cancer. In the present study, single-sample gene set enrichment analysis (ssGSEA) was used to evaluate the expression of cytokines and the degree of immune cell infiltration in four different gastric mucosa tissues from normal gastric mucosa, simple gastritis, and atrophic gastritis to gastric cancer. Here, we show the immune microenvironments of these four gastric mucosae were significantly different. From inflammation to gastric cancer, most immunoinflammatory cells showed a downward trend such as central memory CD4 T cell. Instead, several cells showed an upward trend such as macrophage. Additionally, we found some chemokines/interleukins were illustrated to be low expressed (or highly expressed) in precancerous stage and highly expressed (or low expressed) in postcancerous stage, which demonstrated an opposite expression characteristic in pre-/postcancerous stage.

## 1. Introduction

Gastric mucosal immune microenvironment is mainly generated by the invasion of immune-related cells and cytokines such as chemokines and interleukins in the lamina propria of gastric mucosa under various conditions, which plays an important role in the occurrence and development of diseases such as inflammation and cancer [1, 2]. The immune cells and cytokines in gastric mucosal immune microenvironment appear as a dynamic process along with different gastric diseases [3, 4]. An in-depth understanding of the changes of gastric mucosal immune microenvironment in different disease stages can present us a precise pattern of immune microenvironment in the development of gastritis and gastric cancer, which may provide a valuable theoretical reference and practice guide for early warning, prognosis, and immunotherapy of gastric cancer. Currently, the study for gastric mucosal immune microenvironment was mainly confined to a single stage of disease development. It remains to be thoroughly studied and summarized for the immune microenvironment in various stages from normal gastric mucosa to gastric cancer as well as the dynamic trends of immune cells and cytokines.

In recent years, the rapid development of bioinformatics technology makes it possible to apply public resource of genome expression data to quantitative analysis of immune microenvironment components [5]. Based on the transcriptome data, single-sample gene set enrichment analysis (ssGSEA) focuses on the enrichment score (ES), which is calculated by coordinating the up- or downregulation of genes in a specific sample, by sorting the degree of gene expression in a single sample and integrating the differences among the distribution functions [6]. Therefore, different types of immune cells can be classified and enriched to obtain a quantitative score of different types of immune cells [5].

In the present study, the transcriptome database was integrated from public resource to evaluate the degree of immune cell infiltration and cytokine (chemokines and interleukins) expression in four stages of gastric mucosa tissues from NGM, SG, and AG to GC by ssGSEA and other basic analyses and further to investigate the interaction of immunoinflammatory cells and cytokines/chemokines in the immune microenvironment for different stages of gastric diseases. The study is aimed at providing a theoretical basis for further exploration of immune microenvironment in the progression of gastric diseases and also its clinical significance and application value.

## 2. Methods

### 2.1. Subjects and Information Collection

The endoscopy information and mRNA expression data of 30 patients from Thorell et al. uploaded to ArrayExpress public data source (https://www.ebi.ac.uk/arrayexpress/experiments) in 2017 were used for case data without gastric cancer, including (1) 5 patients with no or very low inflammation sign in the corpus mucosa, (2) 6 patients with gastritis but no atrophic sign in any biopsy specimen, (3) 9 patients with low atrophic gastritis in the corpus, (4) 6 patients with extensive gastric atrophy but no intestinal metaplasia sign, and (5) 4 patients with intestinal metaplasia in the corpus [7] (Table S1). In addition, the information and mRNA expression data of 238 patients in The Cancer Genome Atlas (TCGA) database were selected for case data with gastric cancer (https://portal.gdc.cancer.gov/), which was classified into T1, T2, T3, and T4 stages according to TNM staging: (1) 11 cases in T1 stage (all were intestinal-type gastric cancer), (2) 40 cases in T2 stage (33 cases of intestinal-type gastric cancer and 7 cases of diffuse-type gastric cancer), (3) 127 cases in T3 stage (93 cases of intestinal-type gastric cancer and 34 cases of diffuse-type gastric cancer), and (4) 60 cases in T4 stage (38 cases of intestinal-type gastric cancer and 22 cases of diffuse-type gastric cancer) [8] (Table S1). The above two datasets of patient information and mRNA expression were integrated as the case information for subsequent analyses. The cases were divided into five categories according to the development of gastric disease: normal gastric mucosa (NGM), superficial gastritis (SG), atrophic gastritis (AG), extensive atrophic gastritis with/without intestinal metaplasia (EAG), early and middle stage gastric cancer (T1/T2 stage, T1/2), and advanced gastric cancer (T3/T4 stage, T3/4) (Table S1).

### 2.2. The Normalization of the Data

The voom method is designed to perform linear modeling on RNA-seq data, which use log-counts to normalize the seq data for sequence depth, and then perform the mean-variance trend into a precision weight for each individual normalized observation [9]. Both data of TCGA and Thorell et al. collection are RNA seq data, and we use the voom method to normalize the data from two different resources.

### 2.3. Immune Cell Infiltrated in Different Gastric Mucosa Tissues by ssGSEA Analysis

The mRNA expression data of cases with normal and different gastric diseases and the metagenes of 28 kinds of immune cells were used for ssGSEA analysis [10]. The voom method was used to standardize the raw counts of RNA-seq results from ArrayExpress and TCGA databases [9]. The two datasets of patients' mRNA expression were analyzed using the GSVA package to obtain the infiltration degree of 28 immune cells in all samples [11]. Subsequently, the Mann-Whiney *U* test was adopted to analyze the immune cell infiltration scores in each stage and they were corrected by the Benjamini-Hochberg method.

### 2.4. Expression of Chemokines with Their Receptor and Interleukins in Different Gastric Mucosa by Cluster Analysis

Chemokines with their receptors and interleukins play an important role in inflammatory response. The trends of inflammation in various factors were indirectly evaluated based on the mRNA expression of chemokines with their receptors and interleukins. We combined the two gene datasets of ArrayExpress and TCGA database, extracted 56 kinds of chemokines and chemokine receptors as well as 36 kinds of interleukins (Table S1), and then used hierarchical clustering analysis by calculating the Euclidean distance of samples to cluster the chemokines with their receptors and interleukins. Subsequently, the Mann-Whiney *U* test was employed to analyze the expression of chemokines with their receptors and interleukins in each stage and they were corrected by the Benjamini-Hochberg method.

### 2.5. Correlation among Immunoinflammatory Cells and Chemokines with Their Receptors and Interleukins in Different Gastric Mucosa Tissues by Cross-Linked Analysis

Cross-linked analysis of immune cell infiltration, chemokines with their receptors, and interleukins was performed using Partial Least Squares Regression (PLSR). First, all chemokines were selected as independent variables and an immune infiltrating cell was considered as a dependent variable. Then, the leave-one-out (LOO) cross-validated prediction was used to analyze the data. According to adjCV (bias-corrected CV estimate) minimum, the principal component number was chosen and modeled again using the principal component number. Each chemokine was analyzed by the jackknife estimation, and *p* < 0.05 was adopted for subsequent analyses. By means of the same method, all immunoinfiltrating cells were selected as independent variables and interleukins were regarded as dependent variables. All above-mentioned analyses were performed using the pls package in the R language [12].

## 3. Results

### 3.1. The Results of the Normalized Data

In order to ensure that the expression distribution of each sample is similar in the data from two different sources, we used the voom method for normalization. The box plot is an effective way to reflect whether the distribution of a sample is different from other samples. We use the box plot to show the data distribution after voom processing, and we can see that the distribution of each sample is similar (Figure S1).

### 3.2. Immunoinflammatory Cells Infiltrating in Different Gastric Mucosa Tissues

**Figure 1:**
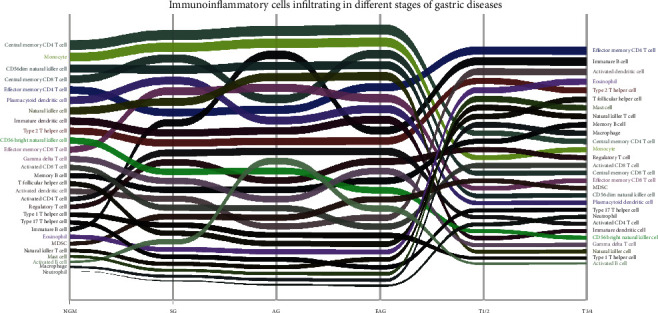
The abundance change of immunoinflammatory cells infiltrating in different stages of gastric diseases. The immune cells with a high ssGSEA score are in the upper part of the graph, and the ones of the low score are in the lower part.

By the ssGSEA method, the infiltration of 28 immune cells was analyzed taking the ssGSEA score as the standard. A higher ssGSEA score indicated more infiltrating immune cells. In normal gastric mucosa, superficial gastritis, atrophic gastritis, and extensive atrophic gastritis with/without intestinal metaplasia, the most infiltrating immune cells were all central memory CD4 T cells and monocytes. In gastric cancer, the most infiltrating immune cells were adaptive immune cells such as effector memory CD4 T cells, immature B cells, and type 2 T helper cells, and innate immune cells such as activated dendritic cells, eosinophils, and mast cells, suggesting a different distribution from those in gastritis. Among gastric cancer, in the early and middle stages (T1/T2 stage, T1/2), the most infiltrating immune cells were effector memory CD4 T cells and immature B cells, which is the same in advanced gastric cancer (T3/T4 stage, T3/4) (Figure 1, Table 1).

**Figure 2:**
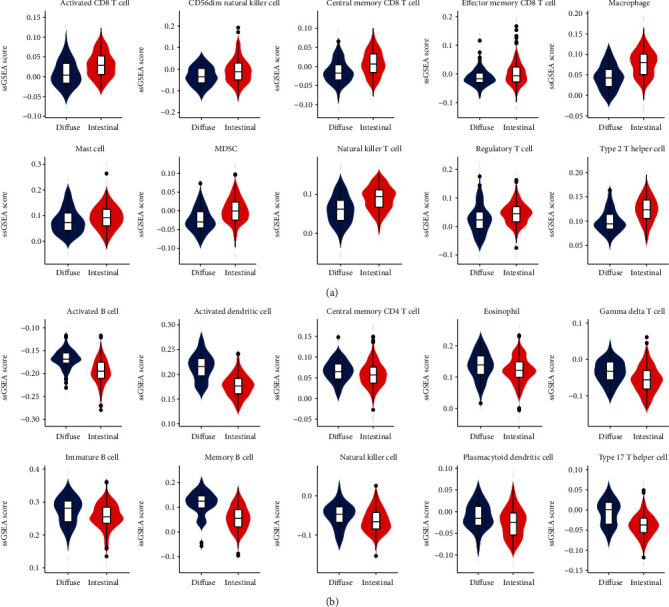
Infiltrating immunoinflammatory cells in different gastric cancer classification. (a) The immune cells with a high degree of infiltration and significant statistical difference in intestinal gastric cancer; (b) the immune cells with a high degree of infiltration and significant statistical difference in diffuse gastric cancer.

Based on the Lauren classification, all gastric cancer cases were divided into intestinal type and diffuse type. The similarities and differences of immune cell infiltration were compared between the two groups. The results showed that the immune cells with a significantly high degree of infiltration in intestinal-type gastric cancer included activated CD8 T cell, CD56dim natural killer cell, central memory CD8 T cell, effector memory CD8 T cell, macrophage, mast cell, MDSC, natural killer T cell, regulatory T cell, and type 2 T helper cell. The immune cells with a significantly high degree of infiltration in diffuse gastric cancer included activated B cell, activated dendritic cell, central memory CD4 T cell, eosinophil, gamma delta T cell, immature B cell, memory B cell, natural killer cell, plasmacytoid dendritic cell, and type 17 T helper cell (Figure 2, Table S2).

### 3.3. Trend of Immunoinflammatory Cells Infiltrating in Different Stages of Gastric Diseases

**Figure 3:**
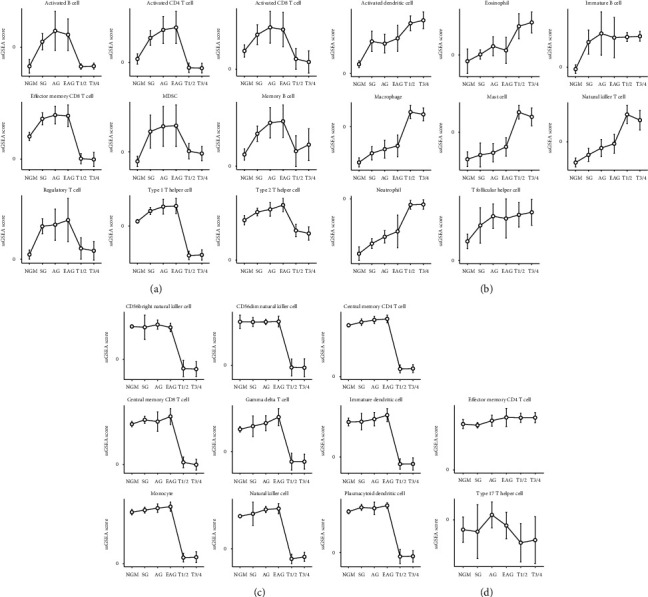
The infiltrating immunoinflammatory cells in different trends along with different gastric diseases, which could be classified into four main status: parabolic type, ascending type, declining type, and stable type. (a) The parabolic type of immune cells; (b) the ascending type of immune cells; (c) the declining type of immune cells; (d) the stable type of immune cells.

During the dynamic process from normal gastric mucosa and gastritis to gastric cancer, the infiltrating immunoinflammatory cells in the lamina propria of gastric mucosa showed different trends along with different gastric diseases, which could be classified into four main status: parabolic type, ascending type, declining type, and stable type. The parabolic type of immune cells included activated B cell, activated CD4 T cell, activated CD8 T cell, effector memory CD8 T cell, MDSC, memory B cell, regulatory T cell, type 1 T helper cell, and type 2 T helper cells, which were common from normal gastric mucosa to superficial, atrophic, and extensive atrophic gastritis till T1/T2 stage gastric cancer (Figure 3(a)). The ascending type of immune cells included activated dendritic cell, eosinophil, mast cell, macrophage, neutrophil, natural killer T cell, immature B cell, and T follicular helper cell, which were common from superficial gastritis to T1/T2 stage gastric cancer, especially from extensive atrophic gastritis to T1/T2 stage gastric cancer, excepting immature B cell (Figure 3(b)). The declining type of immune cells included CD56bright natural killer cell, CD56dim natural killer cell, central memory CD4 T cell, central memory CD8 T cell, gamma delta T cell, immature dendritic cell, monocyte, natural killer cell, and plasmacytoid dendritic cell (Figure 3(c)). The stable type of immune cells included type 17 T helper cells and effector memory CD4 T cell (Figure 3(d)). They were in a relatively stable cell population, which were common from extensive atrophic gastritis to T1/T2 stage gastric cancer (Table S3).

### 3.4. Expression of Chemokines with Their Receptors and Interleukins in Different Gastric Mucosa Tissues

**Figure 4:**
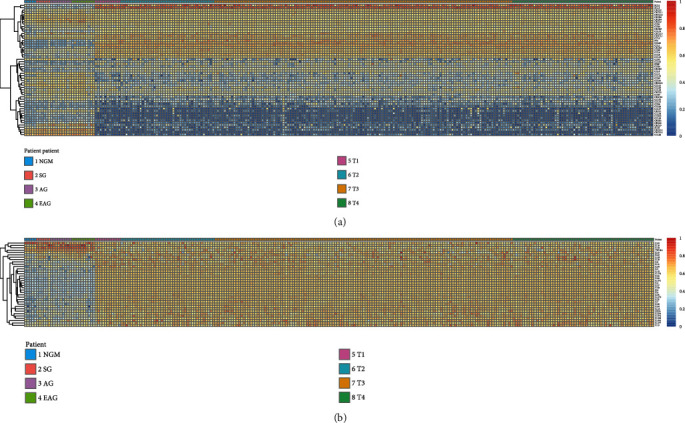
The mRNA expression of interleukins/chemokines and their receptors of different gastric diseases: (a) chemokines and their receptors; (b) interleukins.

Hierarchical clustering was used to analyze the expression of chemokine/chemokine receptors and interleukins in different gastric diseases (Figure 4). In normal gastric tissues, the expression levels of IL33, IL14, CCL28, CXCL14, and CXCL12 were higher than 15 chemokines/interleukins such as IL13 and CXCL6. In superficial gastritis and atrophic gastritis, the expression levels of chemokines/interleukins are relatively the same (IL14, CXCL14, and CCL28 had higher expression levels; IL3, CCL26, IL31, etc., had lower expression levels), only with a slight difference. In gastric cancer tissues, the expression levels of chemokines/interleukins were highly different from those of normal and gastritis immune microenvironment. The top five were XCL1, IL27, IL20, XCL2, and IL7. The lowest five were CCL24, CCL16, CXCL12, CCR10, and CCL17 (Table S4). No significant change was observed in early and midstage as well as advanced gastric cancer. In the intestinal type and diffuse type of gastric cancer, the expression of 28 chemokines with their receptors such as CCL3 and CCL8 and 21 interleukins such as IL7 and IL16 demonstrated significantly statistical difference (Table S5).

### 3.5. The Expression Trend of Chemokines with Their Receptors and Interleukins in Different Stages of Gastric Diseases

Trends of chemokines and interleukins were inextricably linked to the development of immunoinflammatory cells and diseases. It was found that the changes of chemokines and interleukins were mainly concentrated in the transformation stage from atrophic gastritis to the early and midstage of gastric cancer. In this stage, chemokines/interleukins with low or high expression in precancerous stage appeared “reverse expression.” For example, the chemokines XCL2, XCL1, CXCL7, CXCL4, CXCR5, CXCR3, CXCR2, CXCL2, CXCL13, CXCL11, CX3CL1, CCR9, CCR1, CCL8, CCL5, CCL4, CCL3, CCL25, CCL23, CCL14, and CCL13 were lowly expressed in precancerous stage, while in gastric cancer especially the early and middle stages, the expression trend of these lowly expressed chemokines reversed. Similarly, the interleukins IL14, IL33, IL16, IL15, and IL10 had high expression levels in precancerous stage and low expression levels in postcancerous stage (Figure 4, Table S6).

### 3.6. Correlation of Immune Cells with the Expression of Chemokines/Interleukins in Different Gastric Mucosa Tissues at Static Level

**Figure 5:**
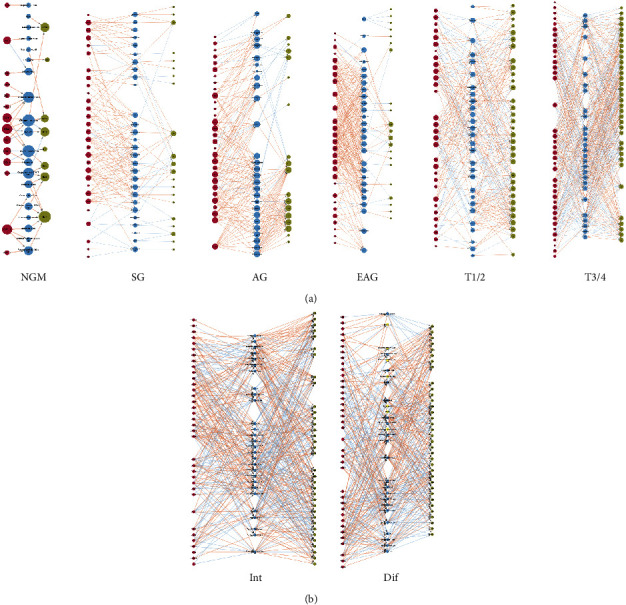
Correlation between the number of immune cells and the expression of cytokines and chemokines in different stages of gastric diseases at static level. Red circles indicate chemokines; blue circles indicate immune cells; grass green circles indicate interleukins. (a) At different stages of gastric diseases (from Min to T3/4); (b) at different Lauren classifications.

By PLSR analysis, we evaluated the correlation among different types of immune cells and chemokines with their receptors and interleukins in the immune microenvironment of different stages of gastric diseases. For example, in normal gastric mucosa, the expression of CX3CL1 and CCL19 was correlated with monocyte infiltration, and the central memory CD8 T cell infiltration was associated with the expression of IL11, IL15, and IL24. In a superficial gastritis environment, the expression of CXCL9, CXCL11, CXCL10, CXCL1, CCL8, CCL5, CCL28, and CCL18 was correlated with central memory CD4 T cell infiltration, and central memory CD8 T cell was associated with IL32 expression. In an atrophic gastritis environment, the expression of CXCL14, CCL4, CCL26, CCL21, CCL2, CCL19, and CCL13 was correlated with the infiltration of central memory CD4 T cell. In extensive atrophic gastritis, the expression of CCL2 and CCL19 was correlated with the infiltration of central memory CD4 T cell, and CD56dim natural killer cell was associated with IL33 expression. CCL5, CCL22, CCL21, CCL2, and CCL15 were correlated with effector memory CD4 T cell in T1/T2 stage gastric cancer, and the number of cells was associated with the expression of IL3, IL17F, IL18, IL22, and IL31. In T3/T4 stage gastric cancer, XCL2, CXCL4, CX3CL1, CCL8, CCL5, CCL3, and CCL25 were correlated with effector memory CD4 T cell, and IL2, IL3, IL12A, IL12B, IL15, IL17C, IL17D, IL18, IL19, IL20, IL22, IL26, IL27, and IL31 were associated with the above correlations (Figure 5(a), Table S7). In the intestinal type and diffuse type of gastric cancer, the correlation was different. In the diffuse type, the expression of XCL1, CXCL7, CX3CL1, CCL8, CCL5, CCL24, CCL21, CCL20, and CCL15 was correlated with immature B cell infiltration, and effector memory CD8 T cell infiltration was associated with the expression of IL17D, IL17F, and IL25. In the intestinal type, however, the expression of XCL2, XCL1, CXCL7, CXCL4, CXCL13, CCL5, CCL25, CCL21, CCL20, CCL2, CCL19, CCL18, CCL17, and CCL15 was correlated with immature B cell infiltration, and effector memory CD8 T cell was associated with the expression of IL1B, IL4, IL5, IL12A, IL17F, IL21, IL24, and IL26 (Figure 5(b), Table S7).

### 3.7. Correlation of Immune Cells with the Expression of Chemokines/Interleukins in Different Stages of Gastric Diseases at Dynamic Level

**Figure 6:**
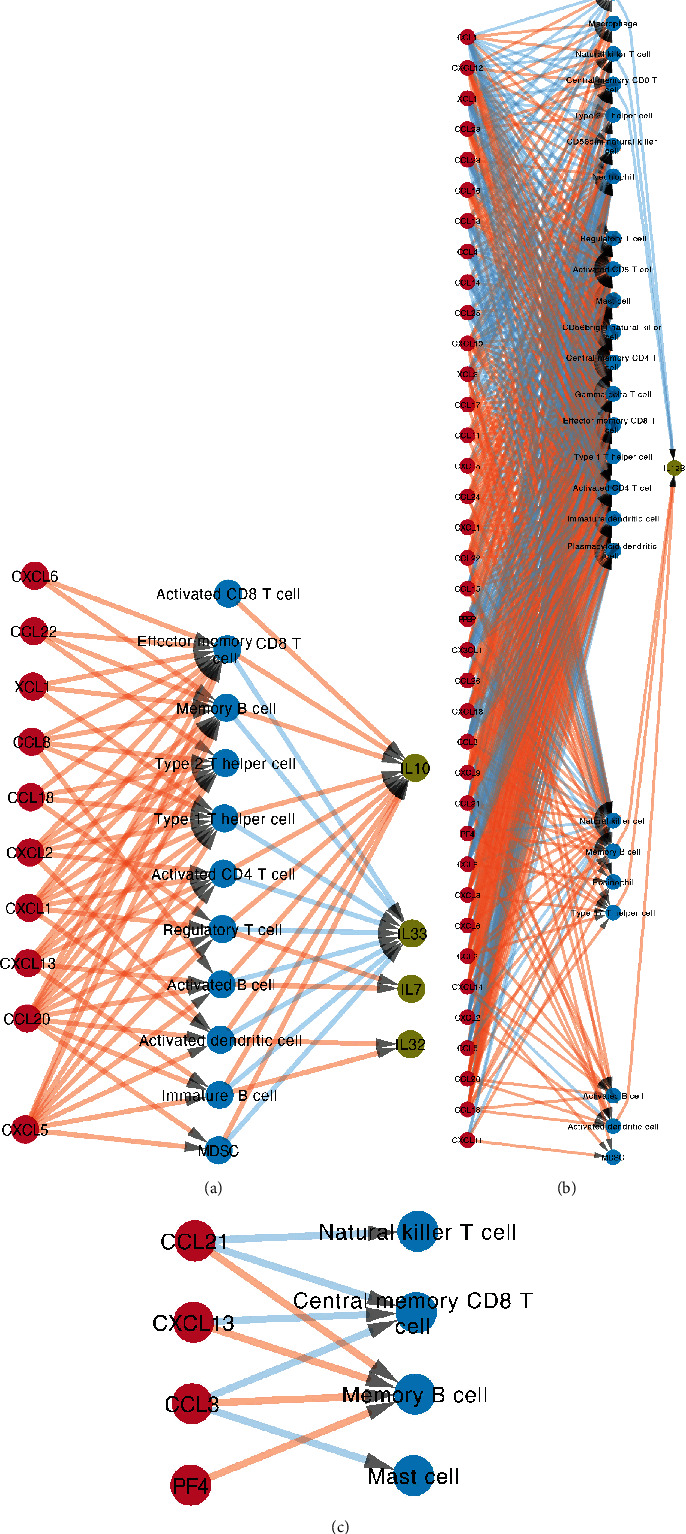
Correlation between the number of immune cells and the expression of cytokines and chemokines in different stages of gastric diseases at dynamic level. Red circles indicate chemokines; blue circles indicate immune cells; grass green circles indicate interleukins, which have statistical significance. (a) From NGM to SG; (b) from EAG to T1/2; (c) from T1/2 to T3/4.

From normal gastric mucosa to superficial gastritis microenvironment, the correlation of immune cells with the expression of chemokines/interleukins was statistically significant. Combined with the findings mentioned above, a total of 78 groups of immune cell infiltration and chemokine/interleukin expression demonstrated changes with statistical significance. Among them, the expression levels of chemokines CXCL5, CXCL13, CCL8, and CCL20 were positively correlated with activated B cell, of which the change of cell infiltration was the highest; the expression levels of IL7 and IL10 were positively correlated with activated B cell infiltration, while the expression level of IL33 was negatively correlated with it (Figure 6(a), Table S8).

579 groups of immune cell infiltration and chemokine/interleukin expression showed statistically significant changes in the immune microenvironment from extensive atrophic gastritis to early and midstage gastric cancer (T1, T2) such as central memory CD4 T cell with the highest change in cell infiltration. The expression of 36 chemokines was associated with central memory CD4 T cell. Among them, the expression of 21 chemokines such as CXCL3 and CCL2 was positively correlated with central memory CD4 T cell, and the other 15 such as CXCL2 and CCL3 had negative correlations. However, no correlation was observed with the expression levels of interleukin (Figure 6(b), Table S8).

No association was found between immune cell and interleukin expression in the immune microenvironment from early and midterm (T1, T2) to advanced (T3, T4) gastric cancer, while some expression changes of chemokines were associated with immune cell. For instance, the expression level of CCL3 was negatively correlated with the changes of mast cells with a more decline in cell number, while the expression level of CXCL4 was positively correlated with the changes of memory B cells with a more elevation cell number (Figure 6(c), Table S8). Regarding the Lauren classification of gastric cancer, the relationship of immune cells with cytokines/chemokines from extensive atrophic gastritis to intestinal- or diffuse-type gastric cancer was not analyzed due to the lacking of clear evidence that extensive atrophic gastritis had to be transformed into some type of the Lauren classification (Figure S2).

## 4. Discussion

This study systematically integrated mRNA-seq data from Thorell et al. collection and TCGA databases to compare the immune microenvironment in different status of gastric mucosa including normal gastric mucosa, superficial gastritis, atrophic gastritis, and gastric cancer. Infiltrating immunoinflammatory cells were quantified by ssGSEA calculation, and chemokines as well as their receptors and interleukins were quantified by cluster classification. The correlation among them was comprehensively analyzed by PLSR comparison at static and dynamic levels, respectively. The results showed that immune microenvironments composed of immune-inflammatory cells and chemokines/interleukins were significantly different in the four stages of gastric mucosa, along with disease progression. To our knowledge, this is the first report on the dynamic analysis of immune microenvironment changes in different gastric diseases. Our study would provide a theoretical basis and evidence support for the role orientation of immune microenvironment during the development of gastric diseases and further its application prospects in clinical diagnosis and treatment of related gastric diseases.

### 4.1. Immune Cells in Different Stages of Gastric Diseases

The type and number of immune cells infiltrated in the lamina propria of gastric mucosa are important components of immune microenvironment. The study suggested that immune cells varied with different gastric diseases. In gastric mucosa, memory T cells act a key role in self-protecting [13]. Our findings further confirmed that when gastric mucosa was under inflammation condition, some immune cells extensively infiltrated, which took a critical part. When the human body is stimulated by antigen, the effector T cells can be resistant to gastric mucosal damage caused by antigen in the early stage of infection. Therefore, a large number of central memory CD4 T cells could be found in gastritis. In addition to T cells, we speculate that innate immune cells such as monocytes may initiate an innate immune response to control infection and secrete corresponding chemokines to induce an adaptive immune response [14]. In gastric cancer, it has been well accepted that effector T cells, NK cells, NKT cells, and mature myeloid dendritic cells can mediate antitumor immunity [15]. Regulatory T cells, B cells, immature myeloid dendritic cells, and plasma cell-like dendritic cells may promote tumor genesis, progression, and growth [15]. Meanwhile, some innate immune cells such as macrophages have a dual-sided effect on tumor development. Here, antitumor immune cells and tumor-promoting immune cells were both found to infiltrate in the mucosa of gastric cancer, suggesting that these immune cells might have a synergistic or antagonistic effect on tumor formation.

Other than the type and number of immunoinflammatory cells, the trend of immunoinflammatory cells in different stages of gastric diseases was also explored. From normal to superficial gastritis, a significant elevation of activated B cells indicated the activation of a humoral immune response [16]. In gastritis, the increase of infiltrating B cells and regulatory T cells simultaneously showed that the body could suppress immune response when fighting with pathogens [17, 18]. Both games and coexistence of immune cells constitute the microenvironment of superficial gastritis.

From superficial gastritis to atrophic gastritis, most cells demonstrated an ascending trend regardless of lacking statistical significance. However, the trends of neutrophil, activated CD4 T cell, and activated B cell were relatively obvious. It was worth noting that although most immune cell infiltration was on the decline from atrophic gastritis to early and midstage gastric cancer (T1, T2 phase), some immune cells presented small change or elevated trend in the degree of infiltration. Despite immune cells did not decrease sharply from normal to inflammatory mucosa in general, a “cliff” decline could be observed from extensive atrophic gastritis to gastric cancer, which might be used as a “marker” to predict gastric cancer.

### 4.2. Chemokines with Their Receptors and Interleukins in Different Stages of Gastric Diseases

We found differences in the expression of chemokines with their receptors in the immune microenvironment of different gastric diseases. In normal gastric mucosa, superficial gastritis, and atrophic gastritis, most chemokines and their receptors were highly expressed, while most interleukins had low expression levels. In gastric cancer, the distribution was almost opposite, in which the interleukins mainly had high expression levels, while the chemokines and their receptors were mainly low expressed. Trends of chemokines and interleukins were inextricably linked to the development of immunoinflammatory cells and diseases [19]. It was found that the changes of chemokines and interleukins were mainly concentrated in the transformation stage from atrophic gastritis to the early and midstage of gastric cancer. In this stage, chemokines/interleukins with low or high expression in precancerous stage appeared “reverse expression.” The cause and mechanism of the “reverse expression” phenomenon are not clear. Further investigations are warranted. Anyway, the findings of “reverse expression” suggest potential application prospects: based on the tendency of reversed expression pattern, we can infer that the disease may be in precancerous to cancerous transformation stage. Real-time monitoring of cytokines and chemokines in patients with atrophic gastritis will provide important clues for the development of gastric cancer. They may also be interfered by corresponding chemokine and interleukin blockers to prevent the expression of tissue in precancerous stage capable for early detection and intervention of gastric cancer.

### 4.3. Correlation of Immune Cells with the Expression of Chemokines/Interleukins in Different Stages of Gastric Diseases

The correlation of immune cells with the expression of chemokines with their receptors and interleukins in different gastric mucosal environments was complex. We focused on the most infiltrating immune cells in different stages of gastric diseases. In normal gastric mucosa, the expression of CX3CL1 and CCL19 was correlated with monocyte infiltration. According to previous researches, CCL19 can promote monocyte adhesion and migration [20], and CX3CL1 has the ability to recruit monocytes, NK cells, CD8+ T cells, and dendritic cells [21]; thus, it is reasonable for the infiltration of monocytes in specific tissue sites under the action of the two chemokines. In superficial and atrophic gastritis, the types of factors and chemokines correlated with central memory CD4 T cell were quite different, which indicated that the chemokines and interleukins recruiting immune cells to the inflammatory tissue varied with the severity of gastritis. These factors exert promotion (positive correlation) or inhibition (negative correlation) effects, which need further exploration. In gastric cancer, the study on effector memory CD4 T cell-related chemokines remains very limited. It has been found that the accumulation of CCL22 in the immune microenvironment of gastric cancer is related to the infiltration of regulatory T cells in gastric cancer [22, 23]. The correlation of CCL22 and effector memory CD4 T cell needs to be confirmed by further experiments. Furthermore, it has also been shown that increased CD4 T cells producing IL22 in tumor tissues are associated with tumor progression and poor prognosis of patients, which is consistent with our results in advanced (T3, T4 phase) gastric cancer [24]. Although our findings cannot be completely explained by previous researches, they are of certain reference value for the exploration of the mechanisms in different stages of gastric cancer progression.

Another highlight of the study is our cross-sectional comparison and correlation analysis for the dynamic trends of immune cells with the expression of chemokines/interleukins. Previous studies have suggested that increased expression of CCL20 and CXCL5 in gastritis tissue can recruit T cells and neutrophils to the gastric mucosal inflammation sites, respectively [25, 26]. This study indicated that the development of gastritis required the collaboration of CXCL5 and CCL20, recruiting not only T cells and neutrophils but also activated B cells. In the inflammation sites, CCL19 or CCL21 can form a dimer with CXCL13 that has a strong chemotactic effect to recruit B cells and memory T cells [27], which is consistent with the increase of activated B cells. Interestingly, other than activated B cells, many immune cells (except Th2 cells, mainly adaptive immune cells) were negatively correlated with IL33. IL33 expression can be detected in normal gastric mucosa, and it becomes higher in patients with asymptomatic gastritis [28]. The expression of IL33 was confirmed to decrease after inflammation. In gastritis, the relationship between IL33 and adaptive immune cells needs further confirmation. At least, however, they were found to have a negative correlation in the progression of gastritis through bioinformatics. Another important process of immune environmental change is from extensive atrophic gastritis to early and midstage gastric cancer. For the positive correlation between chemokines and immune cells, CX3CL1 was associated with four immune cells, and CCL1, CCL3, CCL4, XCL1, and XCL2 were associated with three immune cells. These chemokines may be involved in the transition from inflammation to cancer and play a key role. For the positive correlation between immune cells and cytokines, macrophages, immature B cells, and natural killer T cells were, respectively, associated with IL17, IL15, and IL10, while several other immune cells were associated with 6 interleukins. Therefore, we believed that macrophages took a major part in the immune environment from gastritis to gastric cancer. Under normal conditions, CX3CL1 can recruit monocytes, and then the monocytes accumulated in the tissue can differentiate into macrophages. Hence, the CX3CL1-macrophage axis may exert an important function in the transformation from inflammation to cancer.

## 5. Limitation

The cases analyzed in this study came from two different resources. GC samples in TCGA were collected from gastrectomies, while the case of Dr. Thorell study was collected from endoscopy biopsies. We conducted a further survey on the issue of the different tissue representation from gastrectomies and biopsies. TCGA study uses the DNA/RNA AllPrep Kit (Qiagen) to extract RNA [29], and Dr. Thorell study uses the RNeasy Mini Kit (Qiagen) to extract RNA [7]. The recommended tissue size for both kits is 30 mg [30, 31], so we consider the cases from both sources could reflect the immune microenvironment in the tissue to a certain extent. Even so, as biopsies from endoscopies only reflect a limited view of the immune microenvironment, whether the results of this study fully reflect the true situation needs further verification.

## 6. Conclusion

The present study revealed the microenvironmental characteristics of different gastric diseases composed of immunoinflammatory cells, chemokines, and interleukins, as well as their dynamic changes in the development of gastric cancer. The immunoinflammatory cells were analyzed in different stages of gastric diseases. We also explored the correlation between the chemokine/interleukin trends, differential phenotypes, and potential chemotactic mechanism of immune-inflammatory cells in interstitial infiltration under different gastric diseases including superficial gastritis, atrophic gastritis, and early and advanced gastric cancer. The results showed that the immune microenvironment was significantly different in the four stages of gastric mucosa along with disease progression. The data provides an important theoretical reference for the identification of early diagnostic markers and immunotherapy targets for gastric cancer based on tumor infiltration of immune-inflammatory cells, chemokines, and interleukins.

## Figures and Tables

**Table 1:** The results of immune cell ssGSEA score in different gastric disease stages.

	ssGSEA score					
	NGM (mean ± SD)	SG (mean ± SD)	AG (mean ± SD)	EAG (mean ± SD)	T1/2 (mean ± SD)	T3/4 (mean ± SD)
Immune cell	*n* = 5	*n* = 6	*n* = 9	*n* = 10	*n* = 51	*n* = 187
Activated B cell	−0.1886 ± 0.0677	0.0560 ± 0.0817	0.1638 ± 0.1933	0.1255 ± 0.1570	−0.1911 ± 0.0276	−0.1874 ± 0.0299
Activated CD4 T cell	0.0223 ± 0.0345	0.1635 ± 0.0497	0.2177 ± 0.0914	0.2339 ± 0.1082	−0.0371 ± 0.0327	−0.0397 ± 0.0329
Activated CD8 T cell	0.0574 ± 0.0193	0.1082 ± 0.0313	0.1312 ± 0.0497	0.1243 ± 0.0542	0.0324 ± 0.0326	0.0230 ± 0.0348
Activated dendritic cell	0.0330 ± 0.0123	0.1145 ± 0.0361	0.1065 ± 0.0333	0.1252 ± 0.0461	0.1790 ± 0.0265	0.1895 ± 0.0300
CD56bright natural killer cell	0.1508 ± 0.0072	0.1474 ± 0.0560	0.1592 ± 0.0208	0.1465 ± 0.0197	−0.0411 ± 0.0372	−0.0440 ± 0.0338
CD56dim natural killer cell	0.2835 ± 0.0438	0.2820 ± 0.0299	0.2823 ± 0.0164	0.2848 ± 0.0348	−0.0125 ± 0.0546	−0.0145 ± 0.0575
Central memory CD4 T cell	0.3917 ± 0.0119	0.4155 ± 0.0247	0.4323 ± 0.0268	0.4405 ± 0.0296	0.0583 ± 0.0306	0.0613 ± 0.0302
Central memory CD8 T cell	0.2562 ± 0.0175	0.2833 ± 0.0225	0.2722 ± 0.0616	0.3044 ± 0.0506	0.0139 ± 0.0342	−0.0011 ± 0.0345
Effector memory CD4 T cell	0.2352 ± 0.0232	0.2293 ± 0.0134	0.2526 ± 0.0284	0.2693 ± 0.0464	0.2673 ± 0.0238	0.2682 ± 0.0263
Effector memory CD8 T cell	0.1404 ± 0.0250	0.2499 ± 0.0378	0.2728 ± 0.0549	0.2682 ± 0.0672	0.0029 ± 0.0317	−0.0019 ± 0.0440
Eosinophil	−0.0248 ± 0.0488	0.0020 ± 0.0185	0.0344 ± 0.0380	0.0182 ± 0.0516	0.1138 ± 0.0499	0.1291 ± 0.0418
Gamma delta T cell	0.1089 ± 0.0131	0.1237 ± 0.0506	0.1386 ± 0.0363	0.1682 ± 0.0418	−0.0490 ± 0.0413	−0.0492 ± 0.0364
Immature B cell	−0.0151 ± 0.0373	0.2138 ± 0.1021	0.2867 ± 0.1829	0.2515 ± 0.1711	0.2568 ± 0.0376	0.2633 ± 0.0417
Immature dendritic cell	0.2110 ± 0.0236	0.2128 ± 0.0495	0.2282 ± 0.0411	0.2522 ± 0.0399	−0.0430 ± 0.0315	−0.0421 ± 0.0374
Macrophage	−0.1931 ± 0.0249	−0.1438 ± 0.0359	−0.1215 ± 0.0482	−0.1038 ± 0.0588	0.0786 ± 0.0359	0.0661 ± 0.0350
Mast cell	−0.1473 ± 0.0403	−0.1244 ± 0.0655	−0.1120 ± 0.0502	−0.0799 ± 0.0504	0.1089 ± 0.0372	0.0838 ± 0.0491
MDSC	−0.0457 ± 0.0253	0.0986 ± 0.0803	0.1239 ± 0.0972	0.1271 ± 0.1007	0.0035 ± 0.0380	−0.0081 ± 0.0347
Memory B cell	0.0418 ± 0.0201	0.1163 ± 0.0277	0.1556 ± 0.0548	0.1611 ± 0.0585	0.0537 ± 0.0555	0.0769 ± 0.0572
Monocyte	0.3280 ± 0.0174	0.3412 ± 0.0185	0.3535 ± 0.0274	0.3631 ± 0.0295	0.0384 ± 0.0277	0.0411 ± 0.0352
Natural killer cell	0.2295 ± 0.0099	0.2465 ± 0.0826	0.2763 ± 0.0222	0.2829 ± 0.0355	−0.0710 ± 0.0334	−0.0567 ± 0.0317
Natural killer T cell	−0.0742 ± 0.0161	−0.0469 ± 0.0235	−0.0224 ± 0.0302	−0.0071 ± 0.0344	0.0974 ± 0.0323	0.0772 ± 0.0350
Neutrophil	−0.3628 ± 0.0456	−0.2964 ± 0.0326	−0.2511 ± 0.0413	−0.2153 ± 0.1083	−0.0390 ± 0.0295	−0.0366 ± 0.0312
Plasmacytoid dendritic cell	0.2317 ± 0.0121	0.2553 ± 0.0171	0.2507 ± 0.0352	0.2659 ± 0.0159	−0.0220 ± 0.0403	−0.0209 ± 0.0324
Regulatory T cell	0.0212 ± 0.0207	0.1503 ± 0.0348	0.1577 ± 0.0715	0.1788 ± 0.0989	0.0493 ± 0.0494	0.0385 ± 0.0426
T follicular helper cell	0.0345 ± 0.0130	0.0631 ± 0.0318	0.0798 ± 0.0223	0.0757 ± 0.0352	0.0822 ± 0.0220	0.0868 ± 0.0236
Type 1 T helper cell	0.0196 ± 0.0077	0.0639 ± 0.0151	0.0821 ± 0.0317	0.0848 ± 0.0321	−0.1250 ± 0.0183	−0.1226 ± 0.0218
Type 17 T helper cell	−0.0142 ± 0.0184	−0.0172 ± 0.0393	0.0074 ± 0.0189	−0.0082 ± 0.0194	−0.0332 ± 0.0281	−0.0295 ± 0.0341
Type 2 T helper cell	0.1720 ± 0.0200	0.2071 ± 0.0167	0.2185 ± 0.0296	0.2372 ± 0.0284	0.1276 ± 0.0265	0.1146 ± 0.0285

## Data Availability

The RNA-seq count data that support the findings of this study are available in ArrayExpress under accession number: E-MTAB-3689 and in TCGA database (https://portal.gdc.cancer.gov/).

## Supplementary Materials

Supplementary 1 Figure S1: the normalization of the data using the voom method.

Supplementary 2 Figure S2: correlation between the number of immune cells and the expression of interleukins and chemokines in different stages of gastric diseases at dynamic level. Red circles indicate chemokines; blue circles indicate immune cells; grass green circles indicate interleukins.

Supplementary 3 Table S1: the sample information of E-MTAB-3689 and TCGA-STAD.

Supplementary 4 Table S2: the results of the immune cell ssGSEA score in the Lauren classification of gastric cancer.

Supplementary 5 Table S3: the statistical results of immune cells between different gastric disease stages.

Supplementary 6 Table S4-1: the mRNA expression of chemokines in different gastric disease stages. Table S4-2: the mRNA expression of interleukins in different gastric disease stages.

Supplementary 7 Table S5-1: the mRNA expression of chemokines in the Lauren classification of gastric cancer. Table S5-2: the mRNA expression of interleukins in the Lauren classification of gastric cancer.

Supplementary 8 Table S6-1: the statistical results of chemokines between different gastric disease stages. Table S6-2: the statistical results of interleukins between different gastric disease stages.

Supplementary 9 Table S7-1-1: the results of the correlation between chemokines and immune cells in NGM by PLSR. Table S7-1-2: the results of the correlation between immune cells and interleukins in NGM by PLSR. Table S7-2-1: the results of the correlation between chemokines and immune cells in SG by PLSR. Table S7-2-2: the results of the correlation between immune cells and interleukins in SG by PLSR. Table S7-3-1: the results of the correlation between chemokines and immune cells in AG by PLSR. Table S7-3-2: the results of the correlation between immune cells and interleukins in AG by PLSR. Table S7-4-1: the results of the correlation between chemokines and immune cells in EAG by PLSR. Table S7-4-2: the results of the correlation between immune cells and interleukins in EAG by PLSR. Table S7-5-1: the results of the correlation between chemokines and immune cells in T1/2 by PLSR. Table S7-5-2: the results of the correlation between immune cells and interleukins in T1/2 by PLSR. Table S7-6-1: the results of the correlation between chemokines and immune cells in T3/4 by PLSR. Table S7-6-2: the results of the correlation between immune cells and interleukins in T3/4 by PLSR. Table S7-7-1: the results of the correlation between chemokines and immune cells in diffuse gastric cancer by PLSR. Table S7-7-2: the results of the correlation between immune cells and interleukins in diffuse gastric cancer by PLSR. Table S7-8-1: the results of the correlation between chemokines and immune cells in intestinal gastric cancer by PLSR. Table S7-8-2: the results of the correlation between immune cells and interleukins in intestinal gastric cancer by PLSR.

Supplementary 10 Table S8-1-1: the results of the correlation between chemokines and immune cells from NGM to SG by PLSR. Table S8-1-2: the results of the correlation between immune cells and interleukins from NGM to SG by PLSR. Table S8-2-1: the results of the correlation between chemokines and immune cells from SG to AG by PLSR. Table S8-2-2: the results of the correlation between immune cells and interleukins from SG to AG by PLSR. Table S8-3-1: the results of the correlation between chemokines and immune cells from AG to EAG by PLSR. Table S8-3-2: the results of the correlation between immune cells and interleukins from AG to EAG by PLSR. Table S8-4-1: the results of the correlation between chemokines and immune cells from EAG to T1/2 by PLSR. Table S8-4-2: the results of the correlation between immune cells and interleukins from EAG to T1/2 by PLSR. Table S8-5-1: the results of the correlation between chemokines and immune cells from T1/2 to T3/4 by PLSR. Table S8-5-2: the results of the correlation between immune cells and interleukins from T1/2 to T3/4 by PLSR.
