# Supplementary figures and images for: Infiltration of Immunoinflammatory Cells and Related Chemokine/Interleukin Expression in Different Gastric Immune Microenvironments
Source: J Immunol Res. 2020 Dec 23;2020:2450569. doi: 10.1155/2020/2450569 (PMC7774301; doi:10.1155/2020/2450569)

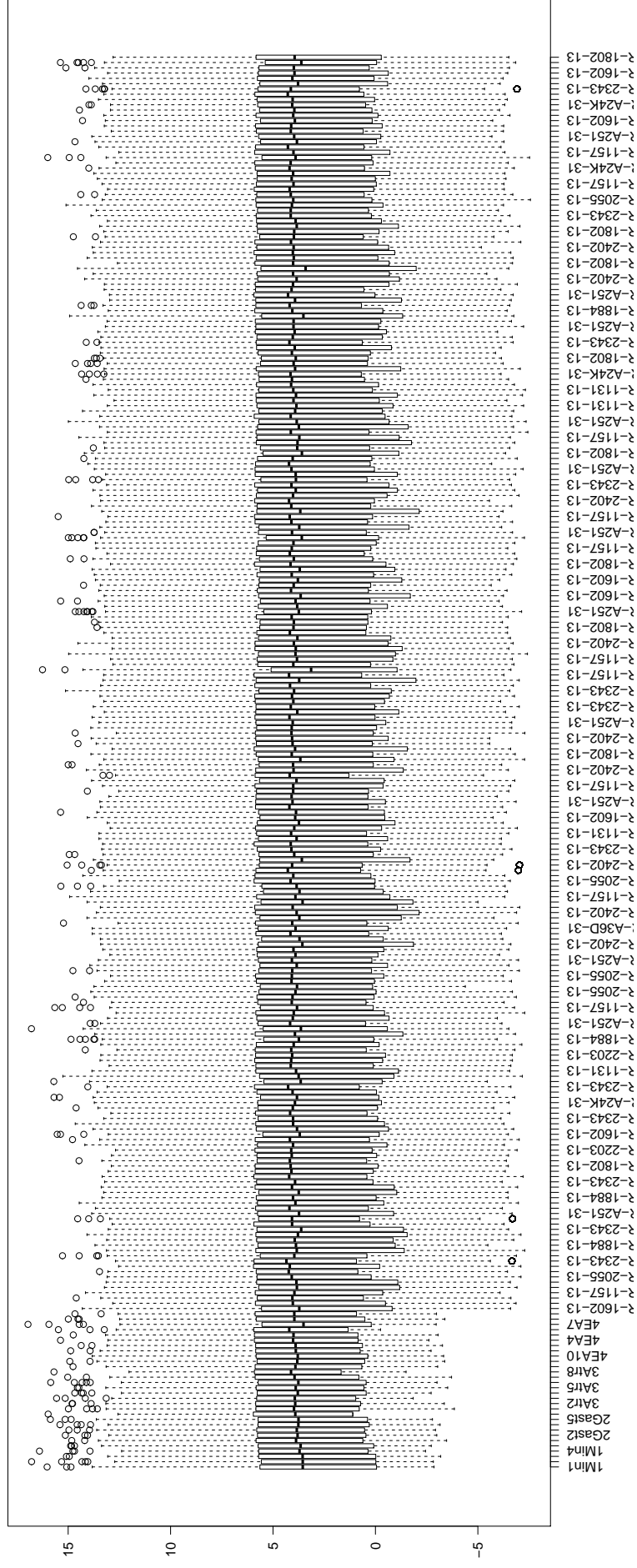

Figure S1. The normalization of the data using voom method.

Supplement: Supplementary 1 — Figure S1: the normalization of the data using the voom method. [file 2450569.f1.pdf]
