# Supplementary material for: Infiltration of Immunoinflammatory Cells and Related Chemokine/Interleukin Expression in Different Gastric Immune Microenvironments
Source: J Immunol Res. 2020 Dec 23;2020:2450569. doi: 10.1155/2020/2450569 (PMC7774301; doi:10.1155/2020/2450569)
Supplement: Supplementary 2 — Figure S2: correlation between the number of immune cells and the expression of interleukins and chemokines in different stages of gastric diseases at dynamic level. Red circles indicate chemokines; blue circles indicate immune cells; grass green circles indicate interleukins. [file 2450569.f2.pdf]

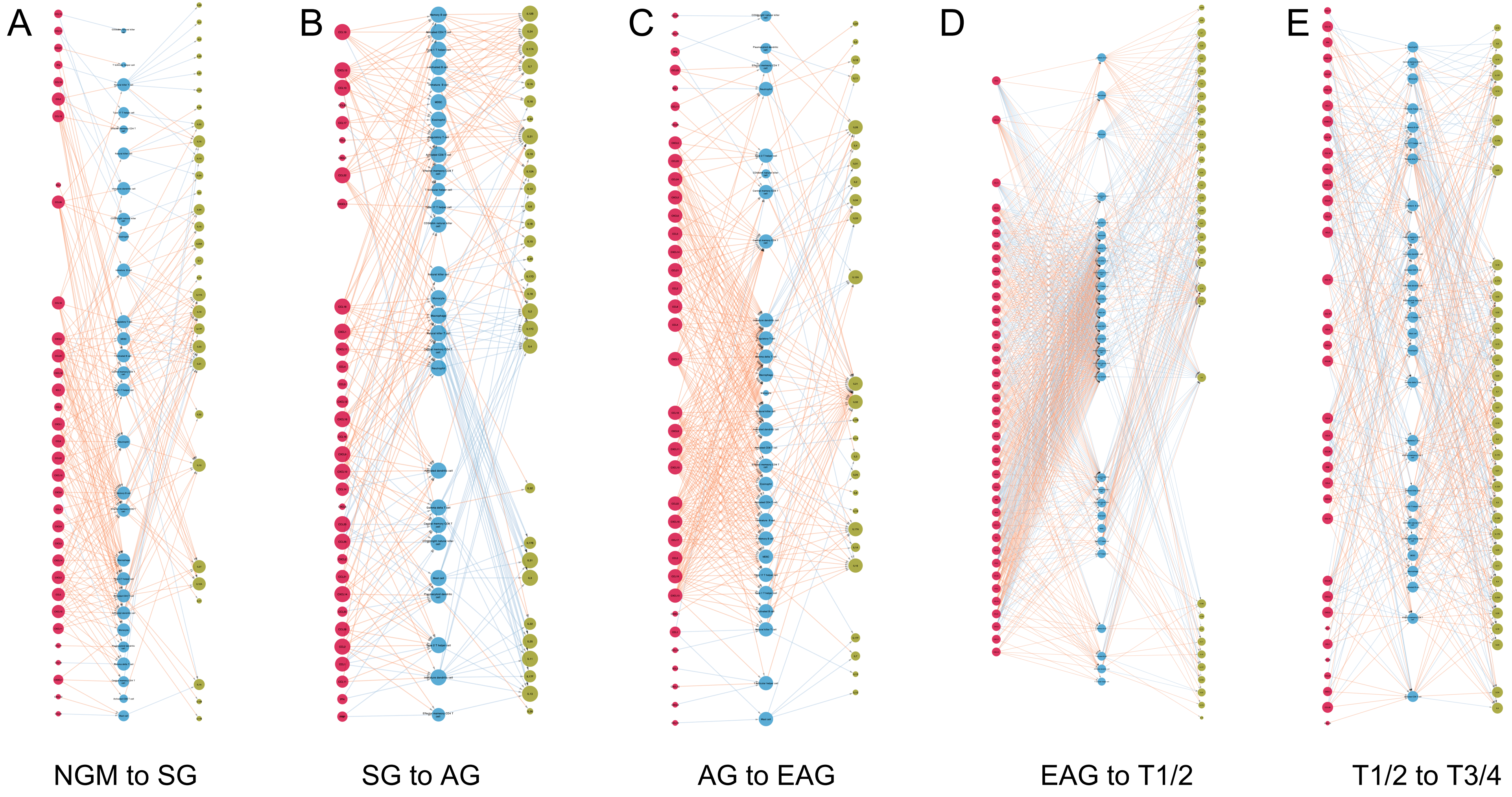

Figure S1. Correlation between the number of immune cells and the expression of interleukins, chemokines in different stages of gastric diseases at dynamic level. Red circles indicate chemokines; blue circles indicate immune cells; grass green circles indicate interleukins. **(A)** From NGM to SG; **(B)** From SG to AG; **(C)** From AG to EAG; **(D)** From EAG to T1/2; **(E)** From T1/2 to T3/4
